# Associations of Free and Reverse Triiodothyronine with Long-Term All-Cause Mortality After Acute Ischemic Stroke and Acute Myocardial Infarction

**DOI:** 10.3390/jcm14051563

**Published:** 2025-02-26

**Authors:** Saulius Taroza, Julius Burkauskas, Aurelija Podlipskytė, Nijolė Kažukauskienė, Narseta Mickuvienė

**Affiliations:** Laboratory of Behavioral Medicine, Neuroscience Institute, Lithuanian University of Health Sciences, LT-00135 Palanga, Lithuania; julius.burkauskas@lsmu.lt (J.B.); aurelija.podlipskyte@lsmu.lt (A.P.); nijole.kazukauskiene@lsmu.lt (N.K.); narseta.mickuviene@lsmu.lt (N.M.)

**Keywords:** free T3, reverse T3, ischemic stroke, myocardial infarction, mortality

## Abstract

**Background:** Arterial thrombosis (AT), the main clinical manifestations of which are ischemic heart disease (IHD) and ischemic stroke (IS), is associated with lowered free triiodothyronine (fT3) in acute ischemic stroke (aIS) and acute myocardial infarction (aMI) but increased reverse T3 (rT3) in aMI, which are associated with worse outcomes at one year. Whether such associations remain independent over a longer follow-up period and the value of rT3 in aIS outcomes are largely unknown. This study was dedicated to examining the impact of fT3 and rT3 on aIS and aMI all-cause mortality over a longer 5-year period. **Methods:** Individuals from Lithuania who experienced aIS and aIM were included in this study. Serum fT3, rT3, free thyroxin and thyroid-stimulating hormone values were examined on admission to the intensive care department. Follow-up for all-cause mortality was divided into two time periods: 1 and 5 years. **Results:** The final study (aIS cohort age, 67.5 ± 9.6 years, 41.5% women and aMI cohort age, 61.8 ± 11.4 years, 28% women) consisted of 241 aIS and 289 aMI individuals, respectively. Lower fT3 was independently associated (OR = 0.41; 95% CI: 0.17–0.99, *p* = 0.049) with aIS, and higher rT3 (OR = 1.69; 95% CI: 1.06–2.67, *p* = 0.027) with aMI with increased all-cause mortality at 1 year. No associations were found between studied hormones and all-cause mortality at 5 years in both conditions. **Conclusions:** Lower fT3 in aIS and higher rT3 in aMI are associated with higher all-cause mortality at 1 year. No such associations were found at 5 years.

## 1. Introduction

Cardiovascular disease (CVD) accounts for one-third of all deaths worldwide [1]. Arterial thrombosis (AT), the main clinical manifestations of which are ischemic heart disease (IHD) and ischemic stroke (IS) [2], tops the list of CVD-associated deaths [1]. Despite the positive developments in the incidence of mortality from CVD, ongoing changes around the world make it necessary to look for measures to help prevent deaths from this disease, especially premature ones [1]. For example, the age-standardized mortality rate due to stroke in Europe has fallen by more than two-thirds in high-income countries and by one-third in middle-income countries over the last three decades [3]. However, an aging and growing population has led to an increased prevalence of stroke and its related total number of deaths in the European Union [4]. Moreover, a significant problem remains the large inequality in stroke and IHD mortality rates among European countries [4].

Acute AT-induced ischemic cell death within the most ischemia-susceptible tissues—the brain and, to a slightly lesser extent, the heart [5]—leads to local tissue loss within minutes and is accompanied by the release of alarmins or damage-associated molecular patterns from dying cells [6,7]. These substances drastically mediate the local and systemic immune response, with consequent interactions involving the autonomic nervous system and neuroendocrine systems resulting in their perturbations [8,9]. Further on, the central parts of the latter two systems can be affected by local brain ischemia and its associated oedema [8]. From the spectrum of major neuroendocrine systems, the thyroid system is receiving increasing attention in CVD reflecting its severity and prognosis [10,11]. The most acute reaction of the thyroid system to illness, recorded even within the first two hours of the onset of acute stress [12], is associated with increased inactivation of the main thyroid gland producing pro-hormone thyroxine (T4) and active hormone triiodothyronine (T3) with concomitant increased production of the T4 metabolite, reverse T3 (rT3), in periphery without changes in thyroid-stimulating hormone (TSH) [13]. This reaction usually manifests with decreased plasma T3 (low-T3 syndrome or low-T3) and increased rT3, and it has a broadly accepted term—nonthyroidal illness syndrome (NTIS) [12]. One of the damaging effects of NTIS on the cardiovascular system could be associated with its deleterious effect on post-ischemic cardiac remodeling [14]; another one could be associated with the progression of heart failure (HF) through the maintenance of fetal gene expression [15]. Furthermore, the detrimental changes caused by low T3 levels during NTIS could be linked to a positive effect of supplementary T3 in HF [16]. In the case of IS, experimental studies in mice showed a beneficial effect of administered T3 on the recovery of neurological function [17].

It is important to note that optimal thyroid hormone (TH) ranges do not match the interval from the 2.5th to 97.5th percentiles, as generally accepted for CVD mortality [18]. Looking more broadly, TH levels, even within the currently attributed euthyroid range, could have an effect on tumorigenesis [19], bone [20] or liver [21] health, among others, and thus a potential impact on mortality due to other or maybe even most causes after experiencing CVD.

There are extensive studies on the relationship between TH and mortality after experiencing CVD. Higher mortality was observed in groups of individuals who experienced acute coronary syndrome (ACS) and had free T3 (fT3) levels below the norm compared to others [22] or in the lowest tertile compared to those in the higher tertiles [23] within 1 and 2.5 years, respectively. Higher mortality after acute IS (aIS) was also associated with belonging to the lowest fT3 tertile compared with the highest one within 1 year [24] and low fT3 compared to the norm within 3 months [25]. With respect to rT3, values higher than the established median were associated with higher 1-year mortality after experiencing acute myocardial infarction (aMI) [26]. It is also important to mention a study with critically ill individuals, including those with CVD, which found that increased rT3 levels were associated with higher mortality during their stay in the intensive care unit, but not with other TH [27], thus showing inconsistencies between studies.

Studies assessing the relationship between mortality after experienced CVD, particularly acute CVD, and TH concentrations, including rT3 levels, over longer follow-up periods (>3 years) are scarce [11]. Thus, our study aimed to explore associations of mortality in individuals without overt thyroid disorder during 1- and 5-year periods after experiencing one of the AT-caused CVD events, aMI or aIS, and their relationship with TH concentrations on admission. It also explored how these findings could be extrapolated between these two different AT-caused events and their consequent mortality.

## 2. Materials and Methods

### 2.1. Study Procedure

This research was part of the Gene–Environment Interactions Connecting Low Triiodothyronine Syndrome and Outcomes of Cardiovascular Disease study [28]. It was carried out in accordance with the regulations of the Declaration of Helsinki and met the specifications of the Regional Biomedical Research Ethics Committee (Permission Numbers: P1-BE-2-11/2013 and P2-BE-2-92 11/2013). The study, performed between 2013 and 2016, involved three different stroke centers (Hospital of the Lithuanian University of Health Sciences, Kauno Klinikos; Klaipėda University Hospital; and Klaipėda Seamen’s Hospital) and one cardiology center (Hospital of the Lithuanian University of Health Sciences, Kauno Klinikos). Individuals with recently experienced aIS, according to the World Health Organization definition [29], and aMI, according to the third universal definition of myocardial infarction (MI) [30], were invited to participate. Confirmation of aIS diagnosis was performed using neuro-imaging, including computed tomography or magnetic resonance imaging. Individuals were included in the study only after their written consent or that of their relatives was obtained and if no ineligibility criteria were present. The following criteria were used to determine ineligibility for inclusion in the study: (1) age outside the range of 18 to 80 years; (2) history of thyroid disorder or recent use of thyroid function-affecting substances (iodinated contrast agent—sudden and high iodide levels could impair thyroid function, resulting in hyperthyroidism or hypothyroidism [31]; Cordarone—could impair thyroid function with TH serum changes through increased iodide release and drug activity on TH deiodination [32]; Carbamazepin—is associated with decreased T3 and T4 levels through effect on increased TH metabolism in liver and competition to thyroid binding globulin [33]; T4 or thyroid-suppressing agent); (3) exhaustion due to malnutrition to prevent confounding with cases with low T3 and concomitant rT3 rise because of nutritional deficit [13]; (4) comorbidity with severe renal or hepatic insufficiency, infection and cancer for prevention of serious conditions leading to higher mortality than CVD itself; (5) arrival at the participating center more than 48 h after the onset of AT—aMI or aIS—event; (6) being outside the TH and TSH limits described below in the section dedicated to the assessment of thyroid profile. Recruitment of individuals is shown in Table 1.

### 2.2. Study Design

The following list of demographic data and CVD risk factors from participants was collected upon arrival at the emergency department of the participating center for further analysis: sex, age, premorbid disability in case of aIS, arterial hypertension (AH), atrial fibrillation (AF), smoking, diabetes mellitus (DM), history of earlier experienced stroke or MI and chronic obstructive pulmonary disease (COPD) in case of aMI. Neurological severity of aIS was assessed using the National Institutes of Health Stroke Scale (NIHSS) [34]. The severity of cardiac damage due to aMI was evaluated according to the Killip classification [35].

Premorbid disability in aIS individuals was assessed using the modified Rankin Scale (mRS) [36]. No or insignificant disability was defined as mRS ≤ 2. AH was assigned based on a positive medical history, use of antihypertensive medications, or a first-time diagnosis of increased blood pressure according to the European Society of Hypertension guidelines [37]. AF was diagnosed based on patient history or irregular RR intervals, as well as absence of distinct P waves in a surface electrocardiogram, in agreement with the European Society of Cardiology recommendations [38]. Active smoking was defined as pre-stroke tobacco smoking behavior or a history of smoking more than one hundred cigarettes ever. DM was assigned if it was previously known or based on a new diagnosis in accordance with the WHO guidelines [39]. Previous stroke and MI were defined based on relevant positive medical history.

Blood samples from participants for TH and TSH evaluation were taken within the first two days after symptom onset upon arrival at the emergency department. Mortality data were gathered from the Causes of Death Register of the Institute of Hygiene under the Ministry of Health of the Republic of Lithuania. The chosen study outcome was all-cause mortality, excluding deaths from external causes, during the first year and during the first five-year period after experiencing aMI or aIS.

### 2.3. Assessment of Thyroid Profile

All included individuals had their serum levels of the TSH, fT3, free T4 (fT4) and rT3 assessed on admission. In the course of laboratory testing, serum was isolated from the blood using centrifugation at 3000× *g* and then frozen at −70 °C according to the Celsius scale. Estimation of the first three hormones serum levels was performed with an electrochemiluminescence immunoassay analyzer (Advia Centaur XP 2016; Siemens Osakeyhtiö, Espoo, Finland), while rT3 was measured using an enzyme immunoassay (ELISA) rT3 kit (EQ 1016-9601-9, EUROIMMUN, Lubeck, Germany). Included individuals had serum hormone levels within the following limits: TSH: 0.1–10 mIU/L (including values within normal limits and subclinical deviations); fT3: ≤4.23 pg/mL (including values within normal limits and lower–for low-T3 cases); fT4: 0.75–1.48 ng/dL (including values within normal limits); and rT3: any value.

A blood sample was collected only once upon arrival at the emergency department, as subsequent radiological examinations were conducted using contrast agents that contained high levels of iodine, which could have distorted the natural activity of deiodinases during the critical condition. The other reasons were associated with a lack of human resources and logistical peculiarities of local hospital facilities.

## 3. Statistics

Statistical calculations were performed using the Statistical Package for the Social Sciences (IBM SPSS, Armonk, NY, USA), version 25. Normally distributed variables were identified using the Kolmogorov–Smirnov test. The latter data were expressed as means ± standard deviations (SDs). Non-normally distributed variables were expressed as medians with interquartile ranges (IQRs). During the analysis, we compared characteristics of individuals who survived and those who passed away within the first year and the first five years using a parametric test (Student’s *t*-test) or its non-parametric equivalent (Mann–Whitney’s U test) according to the data distribution. Associations between evaluated TH, TSH and mortality were assessed by applying univariate regression. Furthermore, during logistic regression fitting, adjustments were made for hormones to account for demographic and vascular risk factors that were significant in univariate analysis. In the group of individuals with aIS, adjustments were made for sex, age, NIHSS and previous stroke for both periods. However, in the aMI group, adjustments were made for sex, age, Killip class, AH and DM during the first year, with additional adjustments for previously experienced stroke and COPD during the first five-year period.

In addition, all hormones tested were included in the multivariate analysis to exclude selection bias that might affect the significance of the results in regression using only one variable [40]. Regression results are reported as the odds ratio (OR) with confidence intervals (CIs). Statistical tests were two-tailed, and the significance level (*p*) was set at less than 0.05.

## 4. Results

Table 2 and Table 3 provide an account of the entry-point characteristics of the 241 and 289 individuals included in this analysis, categorized by all-cause mortality at one and five years after experiencing aIS and aMI, respectively. Groups of non-survivors with aIS within both follow-up periods were older, had more severe neurological impairment according to NIHSS scores, more frequently experienced stroke earlier and had lower fT3 than those who survived. Non-survivors with aMI from both periods were older and had history of AH and DM more often compared to those who survived. Additionally, non-survivors during the first year after aMI more often had a history of previous stroke, COPD and lower fT3 than others. The differences between male and female entry point characteristics of all included individuals with experienced aIS and aMI are shown in the Supplementary Material.

The results of the calculated univariate and multivariate logistic regression analyzing the effect of thyroid function effect on probability of death during both time periods after aIS and aMI are presented in Table 4 and Table 5, respectively. Higher fT3 was associated with lower probability of death during the first year after aIS both in univariate (OR = 0.25; 95% CI: 0.11–0.53; *p* < 0.001) and multivariate (OR = 0.41; 95% CI: 0.17–0.99, *p* = 0.049) analysis (Table 4). The tested thyroid function parameters showed no effect on probability of mortality during the first five years after experienced aIS (Table 4). Higher fT3 was also associated with lower probability of death at one year after aMI in univariate (OR = 0.29; 95% CI: 0.10–0.88; *p* < 0.029) analysis but lost its significance after adjustment (Table 5). Multivariate analysis identified higher rT3 association with higher probability of death at one year after aMI (OR = 1.69; 95% CI: 1.06–2.67, *p* = 0.027) (Table 5). No associations between thyroid function and the probability of death during the first five-year period after aMI were detected (Table 5).

## 5. Discussion

This prospective study assessed the impact of the thyroid profile on long-term all-cause mortality in Lithuanian aIS and aMI individuals over one and five years. The present research established a significant reverse association between fT3 in aIS individuals and mortality at 1 year. In individuals who experienced aMI, a positive association was found after adjustment between rT3 and mortality at one year. No significant associations were found between studied thyroid system hormones and mortality at five years in either condition. The thyroid profile differed for all-cause mortality after aIS and aMI, at least for the one-year prognosis; thus, findings could not be extrapolated from one condition to another. It should be emphasized that the vast majority of the study population consisted of Lithuanian-speaking individuals of Lithuanian origin. The generalizability of the results requires additional studies involving a larger number of subjects, including those of other cultures.

Findings with aIS group individuals associated with increased mortality at one year and lower fT3 are in line with conducted meta-analyses on the impact of thyroid profile at presentation on aIS outcomes. To the best of our knowledge, at least two such meta-analyses were performed, noting that mortality was included into the bad outcomes group but not separately [41,42]. Both research studies had follow-up extending to no more than 1 year and concluded that lower total T3 (tT3) or fT3 was associated with worse aIS outcomes. Looking for the causes of such associations, it is known that NTIS-induced lowered fT3 (within normal limits) or low T3 is a marker of disease severity. However, whether it makes a direct contribution—that lowered fT3 leads to more lesion in clinically euthyroid conditions—needs further investigations [13]. There is no doubt that optimal blood TH levels are necessary for brain tissue functioning during all life courses, but the situation is complicated because every thyroid respondent organ, including the brain, has its own abilities to regulate intra-tissue TH concentrations [43]. Although there are some indications of externally administered neuroprotective T3 action, this needs to be verified clinically [44]. The present study did not find any associations for thyroid profiles with follow-up extended to 5 years. It could be proposed that such a relation was lost because of the increasing importance of other factors, such as functional impairment due to the experienced stroke, contributing to higher mortality over a longer period [45].

Most of the published IHD research linking thyroid profile with its outcomes focuses on the associations of low T3 and, to a lesser extent, rT3 with mortality outcomes. The lack of independent associations for fT3 between survived and non-survived aMI individuals in the performed analysis during both periods could be associated with low number of included individuals and with the low prevalence of low T3 in the studied population. This is also shown by the results of studies, which vary in their conclusions. For example, a study from China that included 1560 individuals with ACS showed just 4% prevalence of low T3 [46], but another from the same country that included 501 aMI individuals found 34.1% of fT3 below the norm [47]. Both studies revealed that low T3 was an independent predictor of all-cause mortality during the first year. We have found just one study on thyroid dysfunction in individuals undergoing coronarography with follow-up extending to 5 years. This study found a higher mortality in NTIS (*n* = 492) compared to euthyroid (*n* = 1045) individuals [48]. Contrary to these investigations, a study from China with 605 individuals with suspected coronary artery disease admitted for coronary angiography found low T3 in 15.7%, even reaching 43.8% in those with severe coronary disease, but did not show significant associations of low T3 with all-cause mortality during the 25 months of follow-up compared to euthyroid individuals [49]. Further on, a study from Sweden that included 331 aMI individuals found no association between tT3 and mortality within the first week and 1 year after the onset of aMI, but a positive association, as in our study, was established with rT3 [26]. Insignificant associations of thyroid profile with mortality, possibly due to the lower number of included cases, were confirmed in an investigation with 70 ACS individuals, where no independent relationships between tT3, fT3, rT3 and mortality were found [50]. Another cause of insignificant associations between fT3 and rT3 (univariate analysis) and all-cause mortality after aMI in our study could be early blood sampling—within the first two days of symptom onset—not capturing their maximal deviations, as it is known that the maximal lowest point of serum tT3 is reached on the third day and the highest for rT3 on the fourth, from the onset of admission to the intensive care unit, because of aMI and unstable angina [51]. In addition, it can be noted that the lack of associations found between the studied TH and mortality after aMI at 5 years was likely due to the impact of other factors on survival that were not assessed during the study or to the subsequent emergence of other conditions. Furthermore, when interpreting the results, it is also important to consider medications not included in the study that were used before and after participants’ inclusion in the research. For example, recently widely used Sodium-Glucose Co-Transport 2 (SGLT2) inhibitors and Glucagon-Like Peptide-1 (GLP-1) receptor agonists have been highly successful in the treatment of diabetes, with an additionally established positive effect on left ventricle function after ACS [52]. Going further, evidence has emerged of possible SGLT2 inhibitors and GLP-1 receptor agonist effects on TSH, but not on fT4 [53,54] or fT3 [53]. The extent to which the use of these or other substances could be associated with the occurrence of NTIS after CVD or the effect on CVD outcomes associated with NTIS warrants future studies.

In NTIS, levels of rT3 increase due to raised T4 peripheral inactivation by the type 3 iodothyronine deiodinase [55]. Previous research provides some evidence about possible rT3 action by non-genomic pathways stimulating proliferation of cancer cells or contribution to vasculopathy in COVID-19 disease [56]. Therefore, it could be speculated that increased rT3 could not only be a marker of disease severity but also act deleteriously, at least in aMI, as was shown in a previous study [26], and contribute to worse outcomes.

From the practical perspective, the results of this research could stimulate a broader assessment of thyroid hormone values in predicting CVD outcomes, especially during the first year after aIS and aMI. Recognition of NTIS in these conditions should guide more intensive monitoring and tailored treatment of these individuals. Although thyroid hormone administration has the potential to improve cardiac function [16], the question of when and in which CVD conditions it would be associated with better outcomes could be answered by future studies.

## 6. Strengths and Limitations

The main strengths of this research consist of data gathered from three different stroke centers and a follow-up of all-cause mortality extended to 5 years.

The limitations consist of a restricted number of included individuals, only one participating center recruiting aMI individuals, blood sampling performed just once and at any time during the 24 h period, the unknown extent of brain and myocardium injury, the unknown rate of contrast agent administration before angiography, the AT recanalization rate, used substances promoting cardiovascular function and the functional status of survivors at the end of their hospital stay. In addition, it is important to note the exclusion of other markers that are important for the prognosis of the CVD, such as echocardiography with innovative myocardial strain indices assessed by speckle tracking echocardiography (STE) in both aIS and aMI individuals. Recent evidence supports the incremental prognostic value of STE-derived parameters for prognostic risk stratification in both aIS [57,58] and aMI [59,60].

## 7. Conclusions

Our research supports an independent negative association of fT3 with all-cause mortality in the aIS group individuals at 1 year, but not at 5 years. In the aMI individuals, an independent positive association was found between rT3 and all-cause mortality within 1 but not 5 years. Further studies with large numbers of subjects are warranted to confirm these data.

## Figures and Tables

**Table 1:** Recruitment of individuals into the study.

aMI Group		aIS Group
N = 405	Potential individuals for enrollment	N = 612
Excluded		
10	Comorbidity with serious condition (infection, liver or renal insufficiency, cancer, severe exhaustion)	58
16	Known thyroid disorder or intake of thyroid-affecting agents	99
15	Thyroid blood testing failed or outside the study enrollment criteria	23
0	Admission >2 days after the onset of symptoms	60
75	Participation refusal	130
0	External causes of death	1
Included into the final analysis		
289		241

aMI, acute myocardial infarction; aIS, acute ischemic stroke.

**Table 2:** Entry point characteristics of all included individuals with experienced acute ischemic stroke and by survival outcomes at two-time intervals.

Entry Point Characteristics	Total	Outcomes at 1 Year		Outcomes at 5 Years	
		Survived	Non-Survived	Survived	Non-Survived
*n*	241	200	41	160	81
Demographic factors					
Age, years, mean ± SD	67.5 ± 9.6	66.3 ± 9.6	73.5 ± 7.0 **^b^**	65.9 ± 9.7	70.7 ± 8.7 **^b^**
Female, *n* (%)	100 (41.5)	80 (40.0)	20 (48.8)	68 (42.5)	32 (39.5)
Male, *n* (%)	141 (58.5)	120 (60.0)	21 (51.2)	92 (57.5)	49 (60.5)
NIHSS score, median (IQR)	8.0 (4.0–13.0)	6.0 (4.0–12.0)	15.0 (9.0–21.0) **^b^**	6.0 (3.0–11.0)	12.0 (6.0–18.0) **^b^**
mRS before AIS ≤ 2, *n* (%)	225 (94.9)	191 (95.5)	34 (91.9)	155 (96.9)	90 (90.9)
Vascular risk factors					
AH, *n* (%)	167 (73.2)	137 (71.7)	30 (81.1)	110 (72.8)	57 (74.0)
AF, *n* (%)	80 (35.1)	63 (33.5)	17 (42.5)	47 (31.8)	33 (41.3)
DM, *n* (%)	35 (15.0)	28 (14.4)	7 (17.9)	22 (14.1)	13 (16.7)
Previous stroke, *n* (%)	50 (20.7)	34 (17.0)	16 (39.0) **^a^**	23 (14.4)	27 (33.3) **^b^**
Previous MI, *n* (%)	24 (10.3)	21 (10.7)	3 (8.1)	17 (10.8)	7 (9.2)
Smoking, *n* (%)	44 (19.3)	39 (20.1)	5 (14.7)	29 (18.7)	15 (20.5)
Thyroid test results on admission					
TSH (mIU/L), median (IQR)	1.19 (0.72–1.96)	1.23 (0.73–1.91)	1.01 (0.52–2.09)	1.22 (0.69–1.86)	1.12 (0.69–2.21)
fT3 (pg/mL), mean ± SD	2.80 ± 0.50	2.85 ± 0.48	2.55 ± 0.52 **^b^**	2.86 ± 0.49	2.68 ± 0.50 **^a^**
Low fT3, *n* (%)	12 (5.0)	7 (3.5)	5 (12.2) **^a^**	5 (3.1)	7 (8.6)
rT3 (ng/mL), mean ± SD	0.34 ± 0.14	0.34 ± 0.13	0.35 ± 0.15	0.35 ± 0.14	0.34 ± 0.13
fT4 (ng/dL), mean ± SD	1.25 ± 0.20	1.25 ± 0.20	1.23 ± 0.20	1.24 ± 0.20	1.26 ± 0.21

AF, atrial fibrillation; AH, arterial hypertension; DM, diabetes mellitus; fT3, free triiodothyronine; fT4, free tetraiodothyronine; IQR, interquartile range; MI, myocardial infarction; mRS, modified Rankin Scale; NIHSS, National Institutes of Health Stroke Scale; SD standard deviation; rT3, reverse triiodothyronine; TSH, thyroid-stimulating hormone; **^a^** *p* < 0.05 compared to non-survived; **^b^** *p* < 0.001 compared to non-survived.

**Table 3:** Entry point characteristics of all included individuals with experienced acute myocardial infarction and by survival outcomes at two-time intervals.

Entry Point Characteristics	Total	Outcomes at 1 Year		Outcomes at 5 Years	
		Survived	Non-Survived	Survived	Non-Survived
*n*	289	259	30	232	57
Demographic factors					
Age, years, mean ± SD	61.8 ± 11.4	61.1 ± 11.1	68.3 ± 11.7 **^b^**	60.6 ± 10.8	67.0 ± 12.1 **^b^**
Female, *n* (%)	81 (28.0)	70 (27.0)	11 (36.7)	61 (26.3)	20 (35.1)
Male, *n* (%)	208 (72.0)	189 (73.0)	19 (63.3)	171 (73.7)	37 (64.9)
ST-elevation aMI, *n* (%)	223 (77.2)	203 (78.4)	20 (66.7)	182 (78.4)	41 (71.9)
Non-ST elevation aMI, *n* (%)	66 (22.8	56 (21.6)	10 (33.3)	50 (21.6)	16 (28.1)
Killip class I, *n* (%)	120 (41.5)	104 (40.2)	16 (53.3)	95 (40.9)	25 (43.9)
Killip class, II *n* (%)	142 (49.1)	131 (50.6)	11 (36.7)	116 (50.0)	26 (45.6)
Killip class III, *n* (%)	3 (1.0)	3 (1.2)	0 (0.0)	3 (1.3)	0 (0.0)
Killip class IV, *n* (%)	24 (8.3)	21 (8.1)	3 (10.0)	18 (7.8)	6 (10.5)
Vascular risk factors					
AH, *n* (%)	237 (82.0)	208 (80.3)	29 (96.7) **^a^**	184 (79.3)	53 (93.0) **^a^**
DM, *n* (%)	55 (19.0)	45 (17.4)	10 (33.3) **^a^**	37 (15.9)	18 (31.6) **^a^**
Previous stroke, *n* (%)	13 (4.5)	9 (3.5)	4 (13.3) **^a^**	8 (3.4)	5 (8.8)
Previous MI, *n* (%)	44 (15.2)	37 (14.3)	7 (23.3)	32 (13.8)	12 (21.1)
COPD, *n* (%)	9 (3.1)	5 (1.9)	4 (13.3) **^a^**	5 (2.2)	4 (7.0)
Thyroid test results on admission					
TSH (mIU/L), median (IQR)	0.99 (0.62–1.54)	0.99 (0.62–1.51)	1.04 (0.50–2.25)	0.98 (0.62–1.51)	10.6 (0.53–1.84)
fT3 (pg/mL), mean ± SD	2.84 ± 0.44	2.86 ± 0.43	2.68 ± 0.48 **^a^**	2.86 ± 0.38	2.79 ± 0.63
Low fT3, *n* (%)	5 (1.7)	3 (1.2)	2 (6.7)	2 (0.9)	3 (5.3)
rT3 (ng/mL), mean ± SD	1.04 ± 0.87	1.02 ± 0.84	1.21 ± 1.07	1.02 ± 0.86	1.13 ± 0.90
fT4 (ng/dL), mean ± SD	1.30 ± 0.21	1.29 ± 0.21	1.31 ± 0.25	1.29 ± 0.20	1.33 ± 0.24

AH, arterial hypertension; aMI, acute myocardial infarction; COPD, chronic obstructive pulmonary disease; DM, diabetes mellitus; fT3, free triiodothyronine; fT4, free tetraiodothyronine; IQR, interquartile range; MI, myocardial infarction; SD standard deviation; rT3, reverse triiodothyronine; TSH, thyroid-stimulating hormone; **^a^** *p* < 0.05 compared to non-survived; **^b^** *p* < 0.001 compared to non-survived.

**Table 4:** Univariate and multivariate logistic regression of associations between thyroid function parameters and mortality over 1 and 5 years after acute ischemic stroke.

Parameters	Univariate Logistic Regression				Multivariate Logistic Regression *		
	OR	95% CI	*p*	R^2^	OR	95% CI	*p*
	Outcomes at 1 year						
TSH, μIU/mL	0.91	0.69–1.20	0.507	0.348	0.91	0.67–1.02	0.568
fT4, ng/dL	0.63	0.12–3.45	0.596		0.96	0.12–7.60	0.966
fT3, pg/mL	0.25	0.11–0.53	<0.001		0.41	0.17–0.99	0.049
rT3, ng/mL	1.74	0.16–19.19	0.652		4.74	0.27–83.08	0.287
	Outcomes at 5 years						
TSH, μIU/mL	1.09	0.90–1.32	0.400	0.256	1.14	0.92–1.41	0.228
fT4, ng/dL	1.40	0.37–5.36	0.839		2.83	0.58–13.95	0.201
fT3, pg/mL	0.46	0.26–0.83	0.009		0.58	0.29–1.16	0.116
rT3, ng/mL	0.58	0.08–4.25	0.590		0.68	0.07–6.64	0.737

fT3, free triiodothyronine; fT4, free tetraiodothyronine; rT3, reverse triiodothyronine; TSH, thyroid-stimulating hormone; * adjusted according to sex, age, NIHSS and previous stroke.

**Table 5:** Univariate and multivariate logistic regression of associations between thyroid function parameters and mortality over 1 and 5 years after acute myocardial infarction.

Parameters	Univariate Logistic Regression				Multivariate Logistic Regression *		
	OR	95% CI	*p*	R^2^	OR	95% CI	*p*
	Outcomes at 1 year						
TSH, μIU/mL	1.58	0.96–2.59	0.071	0.211	1.48	0.85–2.58	0.169
fT4, ng/dL	2.19	0.34–13.99	0.408		2.32	0.29–18.71	0.429
fT3, pg/mL	0.29	0.10–0.88	0.029		0.45	0.14–1.46	0.182
rT3, ng/mL	1.39	0.92–2.08	0.115		1.69	1.06–2.67	0.027
	Outcomes at 5 years						
TSH, μIU/mL	1.23	0.85–1.79	0.276	0.135	1.16	0.77–1.73	0.480
fT4, ng/dL	2.37	0.63–8.85	0.839		1.83	0.42–7.99	0.421
fT3, pg/mL	0.69	0.34–1.40	0.304		0.91	0.43–1.90	0.799
rT3, ng/mL	1.15	0.83–1.57	0.400		1.24	0.88–1.75	0.211

fT3, free triiodothyronine; fT4, free tetraiodothyronine; rT3, reverse triiodothyronine; TSH, thyroid-stimulating hormone; * adjusted according to sex, age, Killip classes, arterial hypertension and diabetes mellitus.

## Data Availability

The raw data supporting the conclusions of this article will be made available by the authors, without undue reservation.

## Supplementary Materials

The following supporting information can be downloaded at: https://www.mdpi.com/article/10.3390/jcm14051563/s1, Table S1. Male and female entry point characteristics of all included individuals with experienced acute ischemic stroke. Table S2. Male and female entry point characteristics of all included individuals with experienced acute myocardial infarction.

## Abbreviations

The following abbreviations are used in this manuscript:

CVD	Cardiovascular disease
AT	Arterial thrombosis
IHD	Ischemic heart disease
IS	Ischemic stroke
T4	Thyroxine
T3	Triiodothyronine
rT3	Reverse triiodothyronine
TSH	Thyroid-stimulating hormone
NTIS	Nonthyroidal illness syndrome
fT3	Free triiodothyronine
aIS	Acute ischemic stroke
aMI	Acute myocardial infarction
MI	Myocardial infarction
AH	Arterial hypertension
AF	Atrial fibrillation
DM	Diabetes mellitus
COPD	Chronic obstructive pulmonary disease
NIHSS	The National Institutes of Health Stroke Scale
fT4	Free thyroxine
ELISA	Enzyme immunoassay
SD	Standard deviation
IQR	Interquartile range
OR	Odds ratio
CI	Confidence intervals
*p*	Significance level
tT3	Total triiodothyronine
HF	Heart failure
STE	Speckle tracking echocardiography
SGLT2	Sodium-Glucose Co-Transport 2 inhibitors
GLP-1	Glucagon-Like Peptide-1 receptor agonists
