# Supplementary material for: Associations of Free and Reverse Triiodothyronine with Long-Term All-Cause Mortality After Acute Ischemic Stroke and Acute Myocardial Infarction
Source: J Clin Med. 2025 Feb 26;14(5):1563. doi: 10.3390/jcm14051563 (PMC11900474; doi:10.3390/jcm14051563)
Supplement: Supplementary file 1 [file jcm-14-01563-s001.zip › jcm-3466534-supplementary.pdf]

Table S1. Male and female entry point characteristics of all included individuals with experienced acute ischemic stroke

Entry point characteristics			p
	**Male**	**Female**	
n	141	100	
**Demographic factors**			
Age, years, mean ± SD	66.2 ± 9.2	69.3 ± 9.0	**0.014**
NIHSS score, median (IQR)	8.0 (4.0–13.0)	7.5 (4.0–14.0)	0.971
mRS before AIS ≤ 2, n (%)	132 (95.0)	93 (94.9)	0.982
Non-survived 1 year	21 (14.9)	20 (20.0)	0.299
Non-survived 5 years	49 (34.8)	32 (32.0)	0.656
**Vascular risk factors**			
AH, n (%)	94 (70.1)	70 (77.7)	0.207
AF, n (%)	43 (32.1)	37 (39.4)	0.257
DM, n (%)	17 (12.5)	18 (18.4)	0.214
Previous stroke, n (%)	25 (17.7)	25 (25.0)	0.170
Previous MI, n (%)	16 (11.8)	8 (8.2)	0.384
Smoking, n (%)	40 (30.3)	4 (4.2)	**<0.001**
**Thyroid test results on admission**			
TSH (mIU/L), median (IQR)	1.04 (0.68–1.68)	1.51 (0.75–2.29)	**0.004**
fT3 (pg/mL), mean ± SD	2.85 ± 0.51	2.72 ± 0.46	**0.028**
Low fT3, n (%)	5 (3.5)	7 (7.0)	0.225
rT3 (ng/mL), mean ± SD	0.34 ± 0.13	0.35 ± 0.14	0.360
fT4 (ng/dL), mean ± SD	1.25 ± 0.20	1.24 ± 0.20	0.630

Table S2. Male and female entry point characteristics of all included individuals with experienced acute myocardial infarction

Entry point characteristics			p
	**Male**	**Female**	
n	208	81	
**Demographic factors**			
Age, years, mean ± SD	59.8 ± 11.4	67.0 ± 9.5	**<0.001**
ST-elevation aMI, n (%)	166 (79.8)	57 (70.4)	0.129
Non-ST elevation aMI, n (%)	42 (20.2)	24 (29.6)	0.129
Killip class I, n (%)	85 (40.9)	43 (43.2)	0.082
Killip class, II n (%)	107 (51.4)	35 (43.2)	0.260
Killip class III, n (%)	1 (0.5)	2 (2.5)	0.432
Killip class IV, n (%)	15 (7.2)	9 (11.1)	0.415
Non-survived 1 year	19 (9.1)	11 (13.6)	0.266
Non-survived 5 years	37 (17.8)	20 (24.7)	0.185
**Vascular risk factors**			
AH, n (%)	164 (78.8)	73 (90.1)	**0.025**
DM, n (%)	30 (14.4)	25 (30.9)	**0.001**
Previous stroke, n (%)	9 (4.3)	4 (4.9)	0.822
Previous MI, n (%)	35 (16.8)	9 (11.1)	0.224
COPD, n (%)	6 (2.9)	3 (3.7)	0.719
**Thyroid test results on admission**			
TSH (mIU/L), median (IQR)	1.00 (0.62–1.54)	0.97 (0.59–1.53)	0.946
fT3 (pg/mL), mean ± SD	2.92 ± 0.43	2.64 ± 0.38	**<0.001**
Low fT3, n (%)	1 (0.5)	4 (4.9)	**0.023**
rT3 (ng/mL), mean ± SD	1.12 ± 0.91	0.84 ± 0.70	**0.005**
fT4 (ng/dL), mean ± SD	1.27 ± 0.19	1.36 ± 0.25	**0.003**
